# A Simple Novel Method for Determining Mortality Rates in HIV Treatment Programs Worldwide

**DOI:** 10.1371/journal.pmed.1000392

**Published:** 2011-01-18

**Authors:** Gregory P. Bisson

**Affiliations:** University of Pennsylvania School of Medicine, Philadelphia, Pennsylvania, United States of America

## Abstract

In this Perspective, Gregory Bisson discusses a new article by Matthias Egger and colleagues that introduces a simple method for HIV treatment programs to more accurately estimate the proportion of patients dying in the first year after ART initiation.

**Linked Research Article**

This Perspective discusses the following new study published in *PLoS Medicine*:

Egger M, Spycher BD, Sidle J, Weigel R, Geng EH, et al. (2011) Correcting mortality for loss to follow-up: A nomogram applied to antiretroviral treatment programmes in sub-Saharan Africa. PLoS Med 8: e1000390. doi:10.1371/journal.pmed.1000390

Matthias Egger and colleagues present a nomogram and a web-based calculator to correct estimates of program-level mortality for loss to follow-up, for use in antiretroviral treatment programs.

UNAIDS and the World Health Organization estimate that in 2009, about 33.3 million people in the world were infected with HIV, of which approximately 5.2 million were on antiretroviral therapy (ART). This proportion amounts to about one-third of those currently considered to need treatment [1],[2]. In many resource-limited settings, rates of loss to follow-up after ART initiation, and of mortality of those lost patients, are high but treatment programs generally do not have the means to actively trace all those who disappear from care [3]–[5]. The combined effect is that, as access to ART is expanded, an increasingly large number of patient outcomes are unknown, many deaths after ART initiation are not counted, and survival within treatment programs is overestimated—at some sites by substantial amounts [6],[7].

For example, if 10,000 patients start ART, 1,000 die during the ensuing year, and all deaths are known to the clinic, the proportion surviving at 1 year post-ART initiation will be correctly calculated as 90%. However, if 500 of these deaths are not discovered, and only known deaths are counted as events, the proportion surviving would be incorrectly estimated as 95%. Why is this of practical interest? At the level of the treatment program, one reason this matters is that a reported 95% survival rate may lead stakeholders to avoid changing delivery of care, whereas a 90% survival rate may trigger more scrutiny and potentially beneficial change. Another programmatic reason is that accurate outcomes data are needed to facilitate comparisons of outcomes in different cohorts to identify treatment approaches that might be either emulated or avoided.

In this issue of *PLoS Medicine,* Matthias Egger and colleagues [8] report a simple method they have created that HIV-treatment programs can use to more accurately estimate the proportion of patients dying in the first year after ART initiation. The method is based on the fact that overall program mortality is a weighted average of mortality among those who remained in care before death (and whose deaths are known) and mortality among those who were lost to follow-up (and whose outcomes are unknown). The authors show that once an estimate of mortality among those who were lost is made (either by tracing a sample of these individuals or by using data from a published meta-regression analysis of outcomes among patients lost to follow-up [4]), then a ratio of mortality among those lost and those remaining in care, and the overall proportion of patients lost to follow-up, can be used to determine a correction factor, *C*. The mortality observed before factoring in deaths among lost patients is then multiplied by *C* and the corrected proportion dying in the first year after ART initiation is obtained. The authors then apply this method to 11 ART programs in sub-Saharan Africa and show that mortality estimates increase from approximately 2% to 10% in absolute terms. While the change before and after correction was minimal for many programs, in one with 28.7% of patients lost to follow-up, the percent dying in the first year increased by nearly 10 times (from 1.3% to 11.2%).

## Is the New Method Too Complex? No.

If the explanation above sounds complex, it does not do justice to the methods. The authors have made a sophisticated approach “field-ready” by creating a nomogram that program managers can use to obtain *C* as long as a few basic parameters about treatment outcomes are known. Nomograms are graphic devices that represent mathematical functions and can be used to very simply enable determination of a third unknown value when two or more other values are known. In this case, if the rate of loss to follow-up, and the ratio of mortality among those lost and not lost to follow-up, are known their values can be marked on a graph and the value of *C* can be obtained by drawing a line. The method should be used only to correct mortality estimates in the first year of ART and, as the authors state, mortality among lost patients will likely change as access to ART expands and patients have more choices of where to access care. Thus, the nomogram will benefit from ongoing input and refinement as global treatment metrics evolve. Nonetheless, the accessibility of the approach is an advance. It is like a point-of-care epidemiologic test for programs.

## Challenges Remain

Perhaps the greatest challenge, however, is that there is no agreement on how to define loss to follow-up, and useful definitions of loss to follow-up should differ depending on whether the goal of identifying such losses is to monitor program-level outcomes or to improve patient care. In the 17 studies evaluated in the meta-regression analysis of mortality among patients lost to follow-up on which the study by Egger et al. is in part based, definitions of loss to follow-up ranged from having missed a visit by 1 week to missing an appointment by 6 months or more [4]. If the goal is to monitor the number of patients within a program who are truly still in care, increasing the number of days late for an appointment required to meet the definition of loss to follow-up increases, to a point, the likelihood that patients so categorized really are lost and will never return [9]. From an epidemiologic perspective it makes sense to use a definition of loss to follow-up that indicates that the lost patient truly is lost. However, another goal of monitoring clinic attendance should be to prevent the adverse outcomes associated with loss to follow-up, including ART discontinuation and death, by addressing the socioeconomic factors associated with missed visits, for example. Such factors have included but are not limited to problems with transportation, work and child-care responsibilities, relocation, fear of disclosure of HIV status or other family barriers, and use of traditional medicines [10],[11]. To the extent that loss to follow-up initiates search efforts, preventing adverse outcomes by monitoring visits will require a definition of loss to follow-up that is more sensitive but less specific, which could translate, for example, into investigations initiated within days, not months, of a missed appointment.

Missed clinic visits are common [9], and while searching for reasons behind each missed visit could waste resources, the role of real-time monitoring of adherence to clinic visits should be aggressively explored, perhaps via use of community health workers and mobile phone technologies, using real-time ART adherence monitoring efforts as a model [12]. In other words, from a patient care perspective, it makes sense to use a definition of loss to follow-up that indicates that the person could still be found.

## Our Understanding of Loss to Follow-Up Grows

Currently we know little about the biology and behaviors that underlie loss to follow-up, but with 5.2 million people on ART, and more starting soon as a result of the 2010 WHO guidelines recommending HIV treatment earlier during disease progression [13], a greater understanding of loss to follow-up in its various forms is needed in order to keep the HIV treatment effort on track. By addressing the effects of loss to follow-up on programmatic mortality estimates, and by providing monitoring efforts with a useful new tool, Egger and colleagues have helped address this need.

## Abbreviations

ART: antiretroviral therapy
